# Early Total Care versus Damage Control: Current Concepts in the Orthopedic Care of Polytrauma Patients

**DOI:** 10.1155/2013/329452

**Published:** 2013-03-21

**Authors:** Ratto Nicola

**Affiliations:** University of Genoa, Largo R. Benzi 10, 16132 Genova, Italy

## Abstract

The management of the polytraumatized orthopedic patient remains a challenging issue. In recent years many efforts have been made to develop rescue techniques and to promote guidelines for the management of these patients. Currently controversies persist between two orthopedic approaches: the Early Total Care and the Damage Control Orthopedics. An overview of the current literature on the orthopedic management of polytrauma patient is provided. Subsequently, femoral shaft fractures, representing extremely common lesions, and pelvic ring injuries, that are associated with a high mortality rate, are analyzed in detail.

## 1. Introduction

The term “polytrauma” is mainly used to describe blunt trauma patients whose injuries involve multiple body regions or cavities, compromise patient's physiology, and potentially cause dysfunction of uninjured organs [1]. Polytrauma is one of the main causes of death in the world. Since young people are frequently involved, trauma is the leading cause of death under the age of 40 [2]. Fractures are frequently components of polytrauma patterns. These lesions must be considered as wounds of bone and soft tissue, giving rise to stress, pain, and hemorrhage. They can be contaminated and cause compartment syndromes with ischemia-reperfusion injury [3]. Patients are at risk of higher morbidity and mortality than the summation of expected morbidity and mortality of each individual injury.

Although polytrauma patients represent a major therapeutic challenge, improved results can be achieved in dedicated institutions with efficient triage and focused trauma specialist care. The treatment of polytrauma patients noted a significant development as a result of better understanding of the physiopathological mechanisms of injury, development of a network of prehospital trauma management, institution of multispecialist integrated groups, and improved intensive care resuscitation. 

The ideal approach to orthopedic injuries is to perform definitive fixation of all fractures in one trip to the operating room. This approach, called Early Total Care (ETC), was widely used in the 80's; not only does it allow the most efficient employment of the operating room and orthopedic surgeons, but also permits patients to be promptly mobilized for tests and therapies [4]. However, there are several scenarios in which immediate definitive fixation of all fractures is not feasible because of patient instability, preventing lengthy operation with associated blood loss. These patients have a primary indication for Damage Control Orthopedics (DCO), a procedure developed since the 90's [5]. Regardless of the preferred approach, a careful evaluation of preoperative patient conditions represents the key factor for selecting the type of initial treatment. 

The aim of this paper is to review the most recent literature on orthopedic polytrauma patient in order to provide a practical flowchart.

## 2. Early Total Care (ETC)

Early stabilization of major skeletal injuries was the mainstay of treatment in trauma surgery in the 80's and early 90's. ETC involves definitive surgical stabilization of all long-bone fractures during the early phase of treatment (24–48 h) [4]. The concept of the ETC holds the merit to focus the attention of the international medical community on the need to stabilize long-bone lesions; this constituted the first step in the development of the modern management of multiple traumas. Previously, these patients were considered “too sick” to undergo surgery, and the manipulation of the fracture stumps was discouraged because of the fear of the so-called “fat embolism syndrome” [6]. In the early 70s, the operative stabilization of femur fractures has been demonstrated to reduce pulmonary complications, promote early patient mobilization and discharge, when compared to traditional nonoperative fracture management [7].

The development of the ETC was made possible by the progressive improvement of osteosynthesis techniques and trauma resuscitation, involving better cardiorespiratory monitoring and the ability to perform prolonged artificial ventilation. In the late 80s, Bone et al. further strengthened this movement with their prospective study, showing the basic role of early surgery. Multi-injured patients treated with ETC appeared to have less pulmonary complications, reduced length of intensive care unit (ICU) and hospital stay (LOS), compared to patients with delayed surgery [8]. 

Even though several studies highlighted this concept and its benefits, opposite views started to emerge during the 90s. ETC was not considered suitable for all polytrauma patients, since in unstable patients it was associated with an unexpectedly high rate of pulmonary complications [9].

## 3. Damage Control Orthopedics (DCO)

The term damage control was originally coined by the US Navy, in reference to keeping afloat a badly damaged ship by procedures to limit flooding, stabilize the vessel, isolate fires and explosions, and avoid their spreading. In abdominal surgery, “damage control” refers to those maneuvers designed to ensure patient survival. It is a staged strategy for the treatment of severe bleeding injury occurring from either blunt or penetrating mechanisms [10]. The same principle, named damage control orthopedics (DCO), was applied to the management of multi-injured patients with long bone and pelvic fractures. It consists of four phases. During the acute phase, life-saving procedures are performed. The priorities of the second phase are the control of hemorrhage, the temporary stabilization of major skeletal fractures, and the management of soft tissue injuries, while minimizing the degree of surgical insult to the patient. Phase three consists of a monitoring period in ICU, while phase four focuses on definitive fracture fixation [11].

The shift from ETC to the DCO came after significant advances in the understanding of pathophysiological and immunological mechanisms regulating the host responses to injury [12]. Traumatic injuries lead to the systemic inflammatory response syndrome (SIRS) followed by a period of recovery mediated by a counter-regulatory anti-inflammatory response (CARS). Severe inflammation may lead to acute organ failure and early death after injury. A mild inflammatory response followed by an excessive CARS may induce a prolonged, deleterious immunosuppressed state. This initial traumatic injury is called the “first hit” and predisposes the patient to a potential risk of deterioration after surgery [13]. In this scenario, surgery may represent the “second hit.” The impact of surgery on the patient biological reserve depends on its type and timing. Fat emboli and hypoxic events, which may result from early surgery, can add damage to the lungs, already injured by pulmonary contusions or rib fractures [14]. 

As far as the emergency orthopedic treatment is concerned, the type of initial stabilization and timing of definitive osteosynthesis modulate these adverse events. External fixation has become the DCO workhorse, because of its rapidity, as well as reduced blood loss and invasiveness. However, less invasive procedures such as splinting and skeletal traction can still play an important role in the initial stabilization of the multi-injured patient. For most upper extremity injuries, simple stabilization with splints or a sling will suffice. For closed fractures below the knee, splinting is usually the best option [15]. For femur fractures, splinting without traction is not effective because the joint above the fracture (the hip) cannot be immobilized. In these fractures, skeletal traction was found to be equal to external fixation in terms of acute respiratory distress syndrome (ARDS), multiple organ dysfunction syndrome (MODS), pneumonia, deep venous thromboembolism (DVT), pulmonary embolism (PE) development, as well as ICU stay, and death rate [16].

Regarding the timing of definitive osteosynthesis, the period-defined “window of opportunity” has been set between the 5th and the 10th days. The posttrauma days 2 to 4 have been reported to be unsuitable for performing definitive osteosynthesis [17]. At this time, sustained immunologic changes are ongoing [18] and fluid shifts, increasing generalized tissue edema, are not yet normalized [19]. In a large survey of over 4000 cases, the effects of the timing of surgery on MODS development were analyzed. Definitive osteosynthesis in patients who later developed MODS was performed between days 2 and 4, whereas patients without MODS were operated on between days 6 and 8 (*P* < 0.0001) [20]. Therefore, the need for a waiting period of several days before definitive osteosynthesis has emerged. However, the waiting period should preferably be shorter than 15 days, since it has been shown that contamination rates in external fixator pin sites rose substantially after 2 weeks [21].

In conclusion, DCO seeks to avoid provoking a severe inflammatory response and confines itself to more modest goals: sufficient stabilization of fractures to prevent further tissue damage and the potential compartment syndrome, while allowing the patient to be mobilized for tests and improved pulmonary care.

## 4. Factors Grading the Clinical Status in Polytrauma

Severely injured patients should be assessed on the basis of the Advanced Trauma Life Support (ATLS) criteria [22]. According to these criteria, a primary survey of airway, breathing, circulation, neurological status, and core temperature is performed. During this phase, the following conditions must be identified and treated: airway obstruction, inadequate ventilation for tension pneumothorax, open pneumothorax, massive hemothorax, mobile chest flap, hemorrhagic shock, or cardiac tamponade.

The next step is based on identification of factors that discourage immediate surgery and lead to selection for DCO. The choice of treatment depends on patient age and comorbidities. The mortality rate is higher in elderly patients [23]. Diabetic patients are subjected to peripheral vascular deficiency and increased risk of limb ischemia following high-energy fracture. Obesity is significantly associated with an increased mortality [24]. Consequently, similar anatomical injuries may lead to different outcomes, based on preexisting patient conditions. Useful prognostic factors for grading the patient clinical status and addressing treatment remain controversial. The first criteria ascertaining the suitability of blunt trauma patients for orthopedic surgery were published in 1978. The authors recommended the use of systolic blood pressure, heart rate, central venous pressure, and hematocrit for basic evaluation. In addition, cardiac index, pulmonary arterial pressure, coagulation status, and acid-base balance were found to be of value during the early period after trauma [25]. Improved knowledge of the pathophysiological mechanisms of trauma allowed identification of four significant clinical factors. Three of them correspond to the so-called lethal triad: hypothermia, coagulopathy, and acidosis [26]. Hypothermia begins at the traumatic insult and is thereafter exacerbated by hypoperfusion, prolonged exposure, and inactivity. Studies have shown that up to 21% of trauma patients are hypothermic at presentation; this figure increases to 46% when patients leave the operating room [27]. Coagulopathy is caused by multiple factors including dilution due to aggressive fluid resuscitation, hypothermia, acidosis, and calcium levels, which have all been shown to affect both the intrinsic and extrinsic clotting cascades. Acidosis is often the result of hemorrhage and shock [11]. Soft tissue injuries are the fourth parameter and may affect the extremities, lung, abdomen, and pelvis. 

Starting from these parameters, Pape described four classes of patients, based on their clinical status: stable, borderline, unstable, and in extremis [26]. A patient is classified into one of these four classes, if he or she meets the criteria in at least three of the four pathophysiological parameters, as reported in Table 1.

Currently, DCO is the preferred approach in “unstable” and “in extremis” patients. In these patients, immediate surgery would be the cause of the “second hit”, which may lead to ARDS, MODS, or even death. Accordingly, DCO should be adopted in patients with a body temperature below 33°C, blood pressure less than 90 mmHg, increased lactate levels, platelets count below 90,000, and major soft tissue injuries [28]. These latter are summarized in Table 1.

Fortunately, the majority of patients fall into the “stable” or “borderline” categories. The ETC is the gold standard in “stable” patients, a condition where subjects have no life-threatening injuries, respond to initial therapy, and are hemodynamically stable without inotropic support. These patients have also no evidence of coagulopathy, ongoing occult hypoperfusion, abnormalities of acid base status, or hypothermia [28]. “Borderline” patients represent the most controversial category in which the choice between ETC and DCO remains uncertain. Pape coined the term “borderline” to describe a patient who is apparently in stable condition before surgery, but who deteriorates unexpectedly and develops organ dysfunction postoperatively [26]. In these patients, the presence of one of the criteria listed in Table 2 is an adverse prognostic factor, which recommends DCO approach. These criteria include the Injury Severity Score (ISS) and specific clinical and radiological findings. The ISS is an anatomical score system that provides an overall score for patients with multiple lesions and correlates with mortality, morbidity, and hospitalization time after trauma. In addition, the hypothesis of a long duration of surgery argues in favor of DCO. Elevated mechanical ventilation time, increased MODS, and mortality rates have been documented after operations exceeding 6 hours, in comparison to shorter operative procedures [29].

Finally, advances in molecular biology might significantly contribute to the development of future therapeutic algorithms. The importance of mediators of the inflammatory response, such as IL-6, IL-8, IL-10, HLA-DR class-II molecules and many other markers, has been recently highlighted [30]. Surgery has been shown to cause a variety of subclinical inflammatory alterations that may result in a cumulative effect as a function of preexisting comorbidity and the time elapsed from trauma to surgery. In polytrauma patients, the recognition of high levels of inflammatory markers could favor the DCO application, in order to prevent the effects of the “second hit” inflammatory reaction. IL-6 is considered the most specific prognostic indicator: early high levels of IL-6 have been associated with the development of organ failure [31]. In fact, IL-6 measurement has already been implemented as a routine laboratory test in several trauma centers.

## 5. Femoral Shaft Fractures

The fixation of femoral shaft fractures (FSF) in polytrauma patients remains a controversial issue, despite the large number of articles published in the last recent decades. In the 70s and 80s several studies demonstrated that ETC of FSF reduces pulmonary complications, mortality, and LOS [8]. Subsequently, this concept was denied by the proponents of the DCO, suggesting that external fixation offers the advantage of early skeletal stability, while minimizing blood loss and anesthesia time of the surgical “second hit” [32, 33]. It was also found that patients with serious abdominal injury and chest trauma benefit the most from delayed treatment [34, 35]. In patients with head injuries, ETC leads to excessive fluid administration, with consequent increased rates of hypoxemia and hypotension, contributing to a poor neurological outcome [36]. 

However, in the recent literature, opposing views emerged, questioning the advantages of the DCO. In 2007, Weninger et al. [37] concluded that early unreamed intramedullary nailing (IMN) of femoral fractures is a safe procedure in multiple injury patients with severe thoracic trauma and seems to be justified to achieve early definitive care. Likewise, Brundage et al. [38] showed that chest and head trauma are not contraindications to early fixation with reamed IMN. In this series of 674 patients with multiple injuries, a relationship between the timing of femur fixation and postoperative pneumonia rate was evident. Femur fixation within 24 hours (*n* = 399), 24 to 48 hours (*n* = 79), 48 to 120 hours (*n* = 23), and greater than 120 hours of injury (*n* = 15) was associated with pneumonia in 15%, 24%, 35% (*P* < 0.05), and 13% of cases, respectively. This demonstrated the relationship between fixation of femur fractures at days 2 to 5 and the development of pulmonary complications.

The conflicting results reported in the literature depend, at least in part, on the different management of multi-injured patients carried out in American and European trauma centers. Reviewing the American literature, O'Toole et al. [39] observed that DCO is applied in only 12% of patients with multiple traumatic injuries and ISS > 17. The rate of DCO performed in American centers [38, 39] is much lower (*P* < 0.05) than in European centers (36% to 47%) [32, 40]. Some European centers aggressively attempt to stabilize femoral fractures in patients with multiple injuries within the first hours after admission [32]. In Pape series [32], only 2% of patients underwent surgery more than 8 hours after admission to the trauma center. This contrasts with American data: 48% of patients with femoral fractures underwent surgical intervention more than 8 hours after admission (average 14 hours) [39]. The differences in the initial management to polytrauma patient with femur fracture may explain the different outcomes experienced by the centers. The European Polytrauma Study Group on the Management of Femur Fractures performed a randomized multicenter study, comparing ETC and DCO. Among “borderline” patients, the incidence of ARDS was 16.7% in the ETC group and 11.1% in the DCO group (*P* = 0.618) [41]. A lower incidence of ARDS is usually reported in North American studies. In the recent North American retrospective series, the incidence of ARDS was 1.5% in the ETC group and 0.0% in the DCO group (*P* = 1.000) [39]. From these data, we may conclude that an adequate resuscitation before surgery is essential.

Finally, bilateral femoral fractures represent a separate entity with different prognosis and therapeutic options. This is a unique scenario associated with a higher mortality and ARDS rates [42]. In addition, these patients have an increased number of associated injuries (up to 80%), thus worsening the prognosis [43]. Although there is a paucity of literature data on this subset of patients, DCO seems to be the ideal strategy.

## 6. Pelvic Ring Injuries

The management of polytrauma patient with pelvic fracture is a major diagnostic and therapeutic challenge both in the immediate postinjury and subsequent definitive fixation phase. Pelvic and acetabular fractures are rare injuries and account for approximately 3% to 8% of all fractures. However, these fractures, often the result of high-energy trauma, are at high risk of associated injuries, which strongly influence the outcome and survival rates [44]. This makes DCO the most suitable therapeutic option for pelvic fracture. 

The poor prognosis of pelvic fractures is related to the high incidence of hemorrhagic shock, due to the anatomical proximity of arteries and veins. Fracture and vascular injury can cause the formation of hematoma in the pelvis and retroperitoneum. This space can hold up to 4 liters of blood before the pressure within the hematoma dabs further hemorrhage [45]. In most of the cases (90%), the bleeding originates from venous disruption or from cancellous bone, while bleeding is due to an arterial injury in only 10% of cases. The mortality of polytrauma patients with pelvic fracture and unstable hemodynamics has been reported to be as high as 50% in one series [46]. Early mortality is usually secondary to uncontrolled hemorrhage, whereas late mortality is due to associated injuries and sepsis-induced MODS. With advances in resuscitation, the mortality directly related to pelvic trauma is most likely closer to 7% [44].

A first classification distinguishes stable fractures (where the pelvic ring is intact or dislocation is minimal and where physical examination did not detect abnormal mobility) from unstable fractures (interruption in the continuity of the pelvic ring with abnormal mobility on physical examination). Fractures are also classified into open or closed depending on whether or not the fracture is in continuity with the skin, the rectum, or the vagina. Open fractures, unstable fractures with displacement greater than 5 mm, and fractures with vertical instability associated with structural lesions of the posterior pelvic ring have a higher risk of bleeding. The Young-Burgess classification is the most widely used, as far as the mechanism of injury, prediction of resuscitation, hemorrhage severity, and associated injuries are concerned. Anteroposterior compression injuries (APC, in which an anteriorly directed force exerts external rotation deformities to the pelvic ring) are associated with the highest mortality and blood transfusion requirements: on average 20% and 14.8 units, respectively. In lateral compression injuries (LC, in which an internal rotation force is directed to the hemi-pelvis) these figures are 7% and 3.6 units, respectively [47].

The decision making in the polytrauma patient with a pelvic ring injury can be divided into two phases: detection and treatment of life-threatening situations (“emergency algorithm”), followed by diagnosis, classification of the osteoligamentous injury, and operative planning, if required. After initial resuscitation according to ATLS protocols, the hemodynamic stability should be immediately evaluated [48]. In fact, resuscitation volume as required in hemodynamically unstable patient is unproductive in the long run without an adequate control of the bleeding site. To quickly identify the source of hemorrhage, the EFAST (extended focused assessment sonography for trauma) technique is nowadays crucial. This ultrasound technique allows a rapid examination of lungs, heart, and abdominal organs in search of the source of bleeding.

In the absence of a clear extrapelvic bleeding (that could explain the hemodynamic instability), the orthopedic surgeon should assume that the cause of the shock is a retroperitoneal hematoma related to the pelvic fracture. At this point, every effort should be aimed at stabilization of the fracture in order to reduce the volume of the open pelvic ring and to dab the venous bleeding. A method that has proved useful over the years is wrapping of the pelvis. This method consists in binding the pelvis with a commercial device, such as the TPOD, or with a sheet, which allows to reduce pelvic volume. This application is rapidly accomplished, is free from side effects, and is usually able to effectively staunch vein bleeding [49]; these patients can then be safely subjected to total-body CT scan. Pelvic binders have largely replaced external fixation and pelvic C-clamp as the best initial means of controlling the hemorrhage associated with unstable fractures of pelvis [50]. In spite of the fact that pelvic external fixation can be rapidly applied, reduces the pelvic volume, and provides temporary fracture stabilization, this fixation is located in front of the patient, while pelvic ring instability is predominantly posterior. By compressing the front, external fixation may widen the posterior pelvis and worsen the problem [51]. The pelvic C-clamp also allows rapid reduction and stabilization of the posterior pelvic ring, through the positioning of two nails in the coccyx and sacroiliac joint. This device does not prevent operators' access to the abdomen but can be burdened with neurological complications, particularly in the presence of sacral fractures [48].

If the patient remains hemodynamically unstable, despite the attempt of external stabilization of the pelvis, the hemorrhagic shock probably has an arterial origin. These patients benefit most from angiographic embolization, a procedure requiring the immediate availability of skilled personnel and highly specialized trauma centers. In a metaanalysis including 26 studies, Karadimas et al. [52] found an overall embolization rate of 8.4% in pelvic fractures. Although, the effectiveness of arteriographic embolization varied from 59%–100% the mortality from pelvic hemorrhage was as high as 25%. Alternatively, pelvic packing is a DCO workable strategy in the presence of pelvic fractures and hemoperitoneum; this approach is especially supported by some European trauma centers. Temporary pelvic packing via laparotomy could significantly aid in pelvic bleeding control and provide crucial time for a more selective management of hemorrhage. However, complication rates were significant, including infection (35%), MODS (9%), and an overall mortality rate of 23% [53]. 

The subsequent definitive fixation can be performed using different techniques depending on the fracture pattern. For example, injuries of the anterior ring are best treated by open reduction and internal fixation with a plate on the symphysis pubis. Disruptions to the sacroiliac ligament can be managed by closed reduction and percutaneous screw fixation, aiming the screw from posterior to anterior across the sacroiliac joint in order to reach the midline of sacrum without emerging anteriorly [54]. In any case, the orthopedic surgeon has to operate only with the final goal of preventing deformities and reducing complications.

ETC is rarely undertaken for the treatment of pelvic ring fractures. However, in the literature, there are data showing benefits from this approach [55]. As previously mentioned, the main benefits result from earlier mobilization, pulmonary rehabilitation, pain control, and therapy.

**Figure 1:**
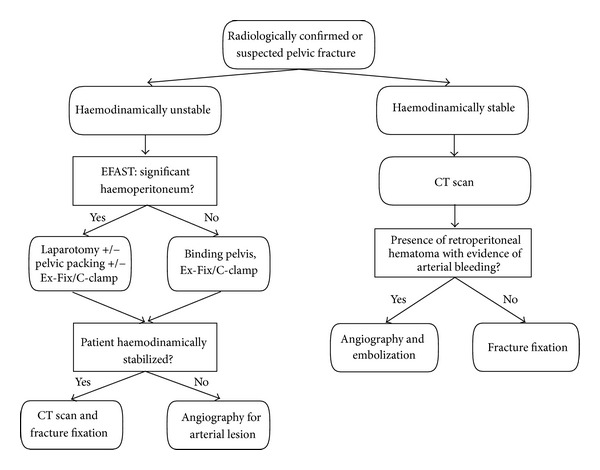
Algorithm representing the management of the pelvic fracture in polytrauma patient. Abbreviations: EFAST: extended focused assessment sonography for trauma; CT Scan: computerized tomography; Ex-Fix: external fixation.

Finally, Figure 1 suggests a possible algorithm that summarizes the previous concepts of pelvic fracture management.

## 7. Conclusion

**Figure 2:**
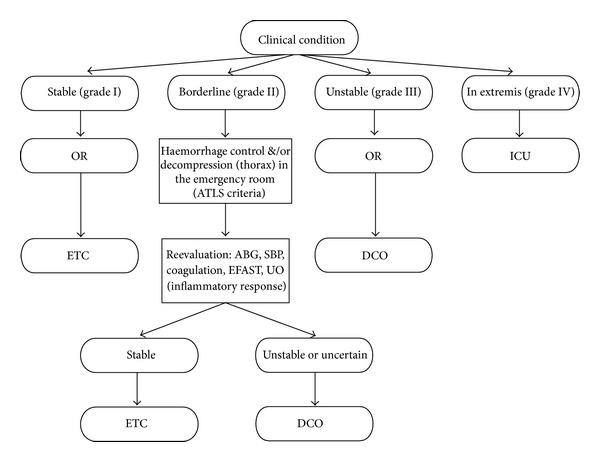
The algorithm for treatment of major fractures, based on patient's clinical categories (data from [26]). Abbreviations: OR: operating room; ICU: intensive care unit; ETC: early total care; DCO: damage control orthopedic; ABG: arterial blood gas; SBP: systolic blood pressure; EFAST: focused assessment with sonography in trauma; UO: urine output.

The variety of clinical presentations in multiple trauma patients makes it difficult to formulate treatment algorithms suitable for each case. The treatment of any individual patient should be tailored according to many variables, such as general medical conditions, fracture patterns, and associated injuries. Grading patients into a range of clinical categories from “stable” to “in extremis” proved to be useful in guiding treatment, as summarized in Figure 2. The treatment of the “borderline” patient represents the most controversial issue. Although DCO reduces the risk of complications related to the “second hit”, it requires a second operation and delays patient mobilization. ETC should be preferred when the clinical condition of the patient and the presence of well-trained surgical teams permit it. For isolated bone lesions, especially the long ones, early stabilization ensures the reduction of complications, resulting in shorter hospitalization and reduced costs. 

Before deciding the type and timing of surgery, efforts must be focused on optimizing ventilatory and hemodynamic parameters and normalizing lactate levels, by means of an adequate resuscitation strategy. The tumultuous progress in the field of molecular biology and genetics is likely to guide future treatment protocols, as suggested by the discovery of a close relationship between blood inflammatory marker levels and the risk of posttraumatic complications. 

## Figures and Tables

**Table 1:** The assessment of the four clinical grades with the corresponding range of clinical parameters (data from [26]).

	Parameter	Stable (grade I)	Borderline (grade II)	Unstable (grade III)	In extremis (grade IV)
Shock	BP (mmHg)	≥100	80–100	60–90	<50–60
	Blood units (2 h)	0–2	2–8	5–15	>15
	Lactate levels	Normal range	Approx 2.5	>2.5	Severe acidosis
	Base deficit (mmol/L)	Normal range	No data	No data	>6–18
	ATLS classification	I	II-III	III-IV	IV
	UO (mL/h)	>150	50–150	<100	<50

Coagulation	Platelet count (*μ*g/mL)	>110000	90000–110000	<70000–90000	<70000
	Factor II and V (%)	90–100	70–80	50–70	<50
	Fibrinogen (g/dL)	>1	Approx 1	<1	DIC
	D-Dimer	Normal range	Abnormal	Abnormal	DIC

Temperature		>35°C	33–35°C	30–32°C	30°C or less

Soft tissue injuries	Lung function, PaO2/FiO2	>350	300	200–300	<200
	Chest trauma scores, AIS	AIS I or II	AIS ≥ 2	AIS ≥ 2	AIS ≥ 3
	TSS	O	I-II	II-III	IV
	Abdominal trauma (moore)	≤II	≤III	III	≥III
	Pelvic trauma (AO classification)	A	B or C	C	C (crush, rollover with abd trauma)
	Extremities	AIS I or II	AIS II-III	AIS III-IV	Crush, rollover, extremities

Abbreviations: BP: blood pressure, ATLS: advanced trauma life support, UO: urine output, TTS: thoracic trauma score, AIS: abbreviated injury scale, DIC: disseminated intravascular coagulation.

**Table 2:** Patient description used for the diagnosis of the “borderline” patient. The presence of any of the parameters is associated with adverse prognosis (data from [26]).

Criteria for the evaluation of “borderline patient”	
Polytrauma ISS 20 and additional thoracic trauma (AIS 2)	
Polytrauma with abdominal/pelvic trauma (Moore 3) and hemodynamic shock (initial blood pressure 90 mm Hg)	
ISS 40 or above in the absence of additional thoracic injury	
Radiographic findings of bilateral lung contusion	
Initial mean pulmonary arterial pressure 24 mm Hg	
Pulmonary artery pressure increases during intramedullary nailing 6 mm Hg	

Abbreviations: ISS: injury severity score, AIS: abbreviated injury scale.
